# Pinching Theorems for Statistical Submanifolds in Sasaki-Like Statistical Space Forms

**DOI:** 10.3390/e20090690

**Published:** 2018-09-11

**Authors:** Ali H. Alkhaldi, Mohd. Aquib, Aliya Naaz Siddiqui, Mohammad Hasan Shahid

**Affiliations:** 1 Department of Mathematics, College of Science, King Khalid University, P.O. Box 9004, Abha 62529, Saudi Arabia; 2 Department of Mathematics, Faculty of Natural Sciences, Jamia Millia Islamia, New Delhi 110025, India

**Keywords:** Casorati curvatures, statistical manifolds, Sasaki-like statistical space forms, *η*-Einstein

## Abstract

In this paper, we obtain the upper bounds for the normalized δ-Casorati curvatures and generalized normalized δ-Casorati curvatures for statistical submanifolds in Sasaki-like statistical manifolds with constant curvature. Further, we discuss the equality case of the inequalities. Moreover, we give the necessary and sufficient condition for a Sasaki-like statistical manifold to be η-Einstein. Finally, we provide the condition under which the metric of Sasaki-like statistical manifolds with constant curvature is a solution of vacuum Einstein field equations.

## 1. Introduction

Information geometry provides a deeper understanding and a geometric approach to families of statistical models. In general, it is related to the study of the differential geometry of statistical manifolds. Information geometry has had a large scope of applications (e.g., physics, chemistry, biology and finance). It has also enabled a joint approach to many problems in the field of differential geometry. The purpose of information geometry is to use tools from Riemannian geometry to extract information from the underlying statistical models. The idea has been successfully used in different areas, including statistical inferences and manifold learning. Amari [1] showed that there are statistical relationships between families of probability densities in terms of the geometric properties of Riemannian manifolds. It is the study of the intrinsic properties of manifolds of probability distributions. In 1989, the notion of statistical submanifolds was introduced and studied by Vos [2]. However, to-date it has made very little progress due to the difficulty in finding classical differential geometric approaches for the study of statistical submanifolds. Furuhata [3] studied hypersurfaces in statistical manifolds and provided some examples as well. In 2006, Takano [4] introduced and studied the statistical structure on Sasakian manifolds, called Sasaki-like statistical manifolds. He also studied Sasaki-like statistical submersions. In 2017, Furuhata et al. [5] gave another notion for the statistical structures on Sasakian manifolds, called Sasakian statistical manifolds, and obtained several results. Recently, some results have been published for statistical manifolds and submanifolds by different geometers [6,7,8,9].

In order to provide an answer to an open question raised by S. S. Chern concerning the existence of minimal immersions into Euclidean spaces of arbitrary dimension, in the early 1990s Prof. B. Y. Chen introduced new types of Riemannian invariants known as Chen invariants or δ-invariants and established general inequalities involving the new intrinsic invariants and the main extrinsic invariant for arbitrary Riemannian manifold. Such invariants and inequalities have many nice applications in several areas of Mathematics. In 1999, Casorati [10] introduced a new extrinsic invariant known as the Casorati curvature. Afterwards, various geometers discussed the geometrical importance of the Casorati curvature [11,12,13]. Due to its geometric importance, a number of results have been obtained in terms of the Casorati curvatures [14,15,16,17,18,19,20,21].

Recently, Lee et al. [19] derived extremities for normalized δ-Casorati curvature for statistical submanifolds in statistical manifold with constant curvature. The purpose of this article is to show that normalized scalar curvature is bounded above by Casorati curvatures for statistical submanifolds in Sasaki-like statistical manifolds of constant ϕ-sectional curvature. Further, we find the condition under which a Sasaki-like statistical manifold becomes η-Einstein, and vice-versa. We also derive the condition which shows that the metric of Sasaki-like statistical manifolds with constant curvature is a solution of vacuum Einstein field equations.

## 2. Sasaki-Like Statistical Manifolds

**Definition** **1.** Let $\left(\bar{N},g\right)$ be a Riemannian manifold and $\bar{\nabla }$ and ${\bar{\nabla }}^{*}$ be torsion-free affine connections on $\bar{N}$. Then, the Riemannian manifold $\left(\bar{N},g\right)$ is said to be statistical if

(1) $$\begin{array}{c} Zg\left(X,Y\right)=g\left({\bar{\nabla }}_{Z}X,Y\right)+g\left(X,{\bar{\nabla }}_{Z}^{*}Y\right), \end{array}$$

*for $X,Y,Z\in \Gamma (T\bar{N})$.*

Here, we remark the following:

1. The connections $\bar{\nabla }$ and ${\bar{\nabla }}^{*}$ are called conjugate connections.
2. If $\left(\bar{\nabla },g\right)$ is a statistical structure on $\bar{N}$, then $\left({\bar{\nabla }}^{*},g\right)$ is also a statistical structure on $\bar{N}$.
3. For the dual connections $\bar{\nabla }$ and ${\bar{\nabla }}^{*}$, we have
  (2) $$\begin{array}{c} {\bar{\nabla }}^{\circ }=\frac{1}{2}\left(\bar{\nabla }+{\bar{\nabla }}^{*}\right), \end{array}$$
  where ${\bar{\nabla }}^{\circ }$ is the Levi-Civita connection for $\bar{N}$.
4. The curvature tensor fields $\bar{R}$ and ${\bar{R}}^{*}$ of $\bar{\nabla }$ and ${\bar{\nabla }}^{*}$, respectively, satisfy
  (3) $$\begin{array}{c} g\left({\bar{R}}^{*}\left(X,Y\right)Z,W\right)=-g\left(Z,\bar{R}\left(X,Y\right)W\right). \end{array}$$

Let $\bar{N}$ be a (2m+1)-dimensional manifold and let N be a *n*-dimensional submanifold of $\bar{N}$. Then, the Gauss formulae are [2]:

(4) $$\begin{array}{c} \left\{\begin{array}{c} {\bar{\nabla }}_{X}Y=\nabla_{X}Y+\zeta \left(X,Y\right),\\ {\bar{\nabla }}_{X}^{*}Y=\nabla_{X}^{*}Y+\zeta^{*}\left(X,Y\right), \end{array}\right. \end{array}$$

where ζ and $\zeta^{*}$ are symmetric, bilinear, imbedding curvature tensors of N in $\bar{N}$ for $\bar{\nabla }$ and ${\bar{\nabla }}^{*}$, respectively. Let us denote the normal bundle of N by $\Gamma (TN^{⊥})$. The linear transformations $A_{N}$ and $A_{N}^{*}$ are defined by

(5) $$\begin{array}{c} \left\{\begin{array}{c} g\left(A_{N}X,Y\right)=g\left(\zeta \left(X,Y\right),N\right),\\ g\left(A_{N}^{*}X,Y\right)=g\left(\zeta^{*}\left(X,Y\right),N\right), \end{array}\right. \end{array}$$

for any $N\in \Gamma (TN^{⊥})$ and X,Y∈Γ(TN). The corresponding Weingarten formulas are [2]:

(6) $$\begin{array}{c} \left\{\begin{array}{c} {\bar{\nabla }}_{X}N=-A_{N}^{*}X+\nabla_{X}^{⊥}N,\\ {\bar{\nabla }}_{X}^{*}N=-A_{N}X+\nabla_{X}^{*⊥}N, \end{array}\right. \end{array}$$

where $N\in \Gamma (TN^{⊥})$, X∈Γ(TN), and $\nabla_{X}^{⊥}$ and $\nabla_{X}^{*⊥}$ are Riemannian dual connections with respect to the induced metric on $\Gamma (TN^{⊥})$.

The corresponding Gauss equations are given by [2]:

(7) $$\begin{array}{ccc} g(\bar{R}\left(X,Y\right)Z,W) & = & g\left(R\left(X,Y\right)Z,W\right)+g\left(\zeta \left(X,Z\right),\zeta^{*}\left(Y,W\right)\right)\\ & - & g(\zeta^{*}\left(X,W\right),\zeta \left(Y,Z\right)) \end{array}$$

and

(8) $$\begin{array}{ccc} g({\bar{R}}^{*}\left(X,Y\right)Z,W) & = & g\left(R^{*}\left(X,Y\right)Z,W\right)+g\left(\zeta^{*}\left(X,Z\right),\zeta \left(Y,W\right)\right)\\ & - & g(\zeta \left(X,W\right),\zeta^{*}\left(Y,Z\right)), \end{array}$$

where R and $R^{*}$ are Riemannian curvature tensors with respect to ∇ and $\nabla^{*}$, respectively.

Let a tensor ϕ be of type (1,1), a vector field ξ, a 1-form η on an odd dimensional manifold $\bar{N}$ satisfying the conditions

$$\begin{array}{c} \eta (\xi )=1,\\ \phi^{2}X=-X+\eta \left(X\right)\xi , \end{array}$$

for any $X\in \Gamma (T\bar{N})$, then we say $\bar{N}$ has an almost contact structure (ϕ,ξ,η).

**Definition** **2** (see Reference [**4**])**.** *A Riemannian manifold $\left(\bar{N},g\right)$ is said to be an almost contact metric-like manifold if it has an almost contact structure (ϕ,ξ,g) on $\bar{N}$ satisfying*

(9) $$\begin{array}{c} g\left(\phi X,Y\right)+g\left(X,\phi^{*}Y\right)=0, \end{array}$$

for $X,Y\in \Gamma (T\bar{N})$, and $\phi^{*}$ is another (1,1) tensor field on $\bar{N}$.

Motivated by Takano’s examples in Reference [4], we provide the following examples:

**Example** **1.** *Let $\bar{N}=\left\{\left(x^{1},\cdots ,x^{m},y^{1},\cdots ,y^{m},z\right)\in R^{2m+1}\right\}$ be a (2m+1)-dimensional semi-Euclidean space which admits the following almost contact metric structure (g,ϕ,ξ,η):*

$$\begin{array}{ccc} \eta & = & \frac{1}{2}\left(dz-\sum_{i=1}^{m}y^{i}dx^{i}\right),\\ \xi & = & -2\partial z,\\ g & = & \frac{1}{4}\left(\eta ⊗\eta +\sum_{i=1}^{m}\left(-2{\left(dx^{i}\right)}^{2}+{\left(dy^{i}\right)}^{2}\right)\right). \end{array}$$

*These equations can be rewritten as follows:*

$$\begin{array}{ccc} \eta & = & \frac{1}{2}\left(-y^{1},-y^{2},\cdots ,-y^{m},0,\cdots 0,1\right),\;\xi =\left(\begin{array}{c} 0\\ \vdots \\ 0\\ -2 \end{array}\right),\\ g & = & \frac{1}{4}\left(\begin{array}{ccc} -2\delta_{ij}+y^{i}y^{j} & 0 & -y^{i}\\ 0 & \delta_{ij} & 0\\ -y^{i} & 0 & 1 \end{array}\right). \end{array}$$

*We also define a tensor field ϕ of type (1,1) in matrix form as:*

$$\begin{array}{ccc} \phi & = & \left(\begin{array}{ccc} 0 & \delta_{ij} & 0\\ -\delta_{ij} & 0 & 0\\ 0 & y^{j} & 0 \end{array}\right). \end{array}$$

We also find another tensor field $\phi^{*}$ of type (1,1) (see Reference [4]):

$$\begin{array}{ccc} \phi^{*} & = & \frac{1}{2}\left(\begin{array}{ccc} 0 & -\delta_{ij} & 0\\ 4\delta_{ij} & 0 & 0\\ 0 & -y^{j} & 0 \end{array}\right). \end{array}$$

*The vector fields $X_{i}=2\partial y^{i}$, $X_{m+i}=2\left(\partial x^{i}+y^{i}\partial z\right)$, and ξ form a ϕ-basis for the contact metric-like structure. Now, we show that*

$$\begin{array}{ccc} g\left(\phi X_{i},X_{m+i}\right)+g\left(X_{i},\phi^{*}X_{m+i}\right) & = & 4g\left(\phi \partial y^{i},\partial x^{i}+y^{i}\partial z\right)+4g\left(\partial y^{i},\phi^{*}\left(\partial x^{i}+y^{i}\partial z\right)\right)\\ & = & 4g\left(\partial x^{i},\partial x^{i}\right)-4g\left(\partial x^{i},y^{i}\partial z\right)+4g\left(\partial y^{i},2\partial y^{i}\right)\\ & = & -2+{\left(y^{i}\right)}^{2}-{\left(y^{i}\right)}^{2}+2\\ & = & 0, \end{array}$$

*and*

$$\begin{array}{ccc} g\left(\phi X_{m+i},X_{i}\right)+g\left(X_{m+i},\phi^{*}X_{i}\right) & = & -4g\left(\partial y^{i},\partial y^{i}\right)-2g\left(\partial x^{i},\partial x^{i}+y^{i}\partial z\right)\\ & = & -1-\frac{1}{2}\left(-2+{\left(y^{i}\right)}^{2}-{\left(y^{i}\right)}^{2}\right)\\ & = & 0. \end{array}$$

*Hence $\left(\bar{N},g,\phi ,\xi ,\eta \right)$ is an almost contact metric-like manifold.*

**Example** **2.** *Consider a 7-dimensional semi-Euclidean space $R^{7}$ endowed with a semi-Riemannian metric g:*

$$\begin{array}{ccc} g & = & \frac{1}{4}\left(\begin{array}{ccc} -2\delta_{ij} & 0 & 0\\ 0 & \delta_{ij} & 0\\ 0 & 0 & 1 \end{array}\right) \end{array}$$

*and the coordinate system $\left(x^{1},x^{2},x^{3},y^{1},y^{2},y^{3},z\right)$. We define ϕ,ξ,η by*

$$\begin{array}{ccc} \phi & = & \left(\begin{array}{ccc} 0 & \delta_{ij} & 0\\ -\delta_{ij} & 0 & 0\\ 0 & 0 & 0 \end{array}\right),\\ \eta & = & \left(0,0,0,0,0,0,\frac{1}{2}\right),\;\xi =-2\left(\begin{array}{c} 0\\ 0\\ 0\\ 0\\ 0\\ 0\\ 1 \end{array}\right). \end{array}$$

*Then, we obtain the following tensor field:*

$$\begin{array}{ccc} \phi^{*} & = & \frac{1}{2}\left(\begin{array}{ccc} 0 & -\delta_{ij} & 0\\ 4\delta_{ij} & 0 & 0\\ 0 & 0 & 0 \end{array}\right). \end{array}$$

*Hence, it is easy to show that $\left(R^{7},g,\phi ,\xi ,\eta \right)$ is an almost contact metric-like manifold.*

**Example** **3.** *Let $R^{9}$ be a 9-dimensional semi-Euclidean space with the standard coordinate $\left(x^{1},x^{2},x^{3},x^{4},y^{1},y^{2},y^{3},y^{4},z\right)$ such that $y^{1}>0,\cdots ,y^{4}>0$. We define the almost contact metric structure (g,ϕ,ξ,η) on $R^{9}$ as follows:*

$$\begin{array}{ccc} g & = & \left(\begin{array}{ccc} \frac{-2}{{\left(y^{i}\right)}^{2}}\delta_{ij} & 0 & 0\\ 0 & \frac{1}{{\left(y^{i}\right)}^{2}}\delta_{ij} & 0\\ 0 & 0 & 1 \end{array}\right),\;\phi =\left(\begin{array}{ccc} 0 & \delta_{ij} & 0\\ -\delta_{ij} & 0 & 0\\ 0 & 0 & 0 \end{array}\right),\\ \xi & = & \left(\begin{array}{c} 0\\ 0\\ 0\\ 0\\ 0\\ 0\\ 0\\ 0\\ 1 \end{array}\right),\;\eta =\left(0,0,0,0,0,0,0,0,1\right). \end{array}$$

*Also, we find*

$$\begin{array}{ccc} \phi^{*} & = & \frac{1}{2}\left(\begin{array}{ccc} 0 & -\delta_{ij} & 0\\ 4\delta_{ij} & 0 & 0\\ 0 & 0 & 0 \end{array}\right). \end{array}$$

*Then we can verify that $\left(R^{9},g,\phi ,\xi ,\eta \right)$ is an almost contact metric-like manifold.*

**Definition** **3** (see Reference [**4**])**.** *A Sasaki-like statistical manifold $\left(\bar{N},\bar{\nabla },g,\phi ,\xi \right)$ is an almost contact metric-like manifold satisfying*

$$\begin{array}{c} {\bar{\nabla }}_{X}\xi =-\phi X,\\ \left({\bar{\nabla }}_{X}\phi \right)Y=g\left(X,Y\right)\xi -\eta \left(Y\right)X,\;X,Y\in \Gamma \left(T\bar{N}\right). \end{array}$$

**Example** **4.** *Let $R^{5}$ be a 5-dimensional semi-Euclidean space with local coordinate system $\left(x^{1},x^{2},y^{1},y^{2},z\right)$. We put the following almost contact metric structure (g,ϕ,ξ,η) on $R^{5}$:*

$$\begin{array}{ccc} g & = & \frac{1}{4}\left(\begin{array}{ccc} 2\delta_{ij} & 0 & 0\\ 0 & -\delta_{ij} & 0\\ 0 & 0 & 1 \end{array}\right),\;\phi =\left(\begin{array}{ccc} 0 & \delta_{ij} & 0\\ -\delta_{ij} & 0 & 0\\ 0 & 0 & 0 \end{array}\right),\\ \xi & = & \left(\begin{array}{c} 0\\ 0\\ 0\\ 0\\ 1 \end{array}\right),\;\eta =\left(0,0,0,0,1\right). \end{array}$$

*Another tensor field of type (1,1) is given by*

$$\begin{array}{ccc} \phi^{*} & = & \frac{1}{2}\left(\begin{array}{ccc} 0 & -\delta_{ij} & 0\\ 4\delta_{ij} & 0 & 0\\ 0 & 0 & 0 \end{array}\right). \end{array}$$

The dual affine connections $\bar{\nabla }$ and ${\bar{\nabla }}^{*}$ are defined as

$$\begin{array}{c} {\bar{\nabla }}_{\partial x^{i}}\partial x^{j}={\bar{\nabla }}_{\partial y^{i}}\partial y^{j}={\bar{\nabla }}_{\partial z}\partial z=0,\\ {\bar{\nabla }}_{\partial x^{i}}\partial y^{j}={\bar{\nabla }}_{\partial y^{j}}\partial x^{i}=-2\delta_{ij}\partial z,\\ {\bar{\nabla }}_{\partial x^{i}}\partial z={\bar{\nabla }}_{\partial z}\partial x^{i}=\partial y^{i}=-\phi \partial x^{i},\\ {\bar{\nabla }}_{\partial y^{i}}\partial z={\bar{\nabla }}_{\partial z}\partial y^{i}=-\partial x^{i}=-\phi \partial y^{i},\\ {\bar{\nabla }}_{\partial x^{i}}^{*}\partial x^{j}={\bar{\nabla }}_{\partial y^{i}}^{*}\partial y^{j}={\bar{\nabla }}_{\partial z}^{*}\partial z=0,\\ {\bar{\nabla }}_{\partial x^{i}}^{*}\partial y^{j}={\bar{\nabla }}_{\partial y^{j}}^{*}\partial x^{i}=\delta_{ij}\partial z,\\ {\bar{\nabla }}_{\partial x^{i}}^{*}\partial z={\bar{\nabla }}_{\partial z}^{*}\partial x^{i}=-2\partial y^{i}=-\phi^{*}\partial x^{i},\\ {\bar{\nabla }}_{\partial y^{i}}^{*}\partial z={\bar{\nabla }}_{\partial z}^{*}\partial y^{i}=\frac{1}{2}\partial x^{i}=-\phi^{*}\partial y^{i}, \end{array}$$

*for i,j=1,2. We compute the following:*

(10) $$\begin{array}{ccc} \left({\bar{\nabla }}_{\partial x^{i}}\phi \right)\partial x^{j} & = & {\bar{\nabla }}_{\partial x^{i}}\phi \partial x^{j}-\phi {\bar{\nabla }}_{\partial x^{i}}\partial x^{j}\\ & = & -{\bar{\nabla }}_{\partial x^{i}}\partial y^{j}\\ & = & 2\delta_{ij}\partial z, \end{array}$$

*and*

(11) $$\begin{array}{ccc} g\left(\partial x^{i},\partial x^{j}\right)\partial z-\eta \left(\partial x^{j}\right)\partial x^{i} & = & 2\delta_{ij}\partial z-g\left(\partial x^{j},\partial z\right)\partial x^{i}\\ & = & 2\delta_{ij}\partial z. \end{array}$$

*From Equations (10) and (11), we conclude that*

$$\begin{array}{c} \left({\bar{\nabla }}_{\partial x^{i}}\phi \right)\partial x^{j}=g\left(\partial x^{i},\partial x^{j}\right)\partial z-\eta \left(\partial x^{j}\right)\partial x^{i}. \end{array}$$

*Thus, we say that $\left(R^{5},\bar{\nabla },g,\phi ,\xi ,\eta \right)$ is a Sasaki-like statistical manifold.*

**Definition** **4** (see Reference [**4**])**.** *Let $\left(\bar{N},\bar{\nabla },g,\phi ,\xi \right)$ be Sasaki-like statistical manifold and c∈R. The Sasaki-like statistical manifold is said to be of constant ϕ-sectional curvature c if $\bar{R}$ is defined by*

(12) $$\begin{array}{cc} \bar{R}\left(X,Y\right)Z & =\frac{c+3}{4}\left\{g\left(Y,Z\right)X-g\left(X,Z\right)Y\right\}\\ & +\frac{c-1}{4}\{g\left(\phi Y,Z\right)\phi X-g\left(\phi X,Z\right)\phi Y\\ & -g(\phi X,Y)\phi Z+g(X,\phi Y)\phi Z-g(Y,\xi )g(Z,\xi )X\\ & +g(X,\xi )g(Z,\xi )Y+g(Y,\xi )g(Z,X)\xi -g(X,\xi )g(Z,Y)\xi \}, \end{array}$$

*where $X,Y,Z\in T\bar{N}$. We denote a Sasaki-like statistical manifold with constant ϕ-sectional curvature c by $\bar{N}\left(c\right)$.*

**Remark** **1.** The curvature tensor ${\bar{R}}^{*}$ can also be obtained by changing ϕ for $\phi^{*}$ in Equation (12).

**Definition** **5** (see Reference [**22**])**.** *Let $\bar{N}$ be a contact metric manifold. Then, $\bar{N}$ is said to be an η-Einstein if its Ricci tensor S has the following form:*

(13) $$\begin{array}{c} S=\mu g+\nu \eta ⊗\eta , \end{array}$$

*for any smooth function μ and ν on $\bar{N}$. Moreover, if the function ν=0 in Equation (13), then the manifold $\bar{N}$ becomes Einstein, and if both the functions μ and ν vanish in Equation (13), then the manifolds are known as Ricci-flat manifolds.*

Let $\left\{e_{1},\cdots ,e_{n},e_{n+1}=\xi \right\}$ and $\left\{e_{n+2},\cdots ,e_{2m+1}\right\}$ be the tangent orthonormal frame and normal orthonormal frame, respectively, on N. The mean curvature vector fields are given by

(14) $$\begin{array}{c} H=\frac{1}{n}\sum_{i=1}^{n+1}\zeta \left(e_{i},e_{i}\right), \end{array}$$

(15) $$\begin{array}{c} H^{*}=\frac{1}{n}\sum_{i=1}^{n+1}\zeta^{*}\left(e_{i},e_{i}\right), \end{array}$$

and

(16) $$\begin{array}{c} H^{\circ }=\frac{1}{n}\sum_{i=1}^{n+1}\zeta^{\circ }\left(e_{i},e_{i}\right). \end{array}$$

We also set

(17) $$\begin{array}{c} {∥\zeta ∥}^{2}=\sum_{i,j=1}^{n+1}g\left(\zeta \left(e_{i},e_{j}\right),\zeta \left(e_{i},e_{j}\right)\right), \end{array}$$

(18) $$\begin{array}{c} ∥\zeta^{*}{∥}^{2}=\sum_{i,j=1}^{n+1}g\left(\zeta^{*}\left(e_{i},e_{j}\right),\zeta^{*}\left(e_{i},e_{j}\right)\right), \end{array}$$

and

(19) $$\begin{array}{c} ∥\zeta^{\circ }{∥}^{2}=\sum_{i,j=1}^{n+1}g\left(\zeta^{\circ }\left(e_{i},e_{j}\right),\zeta^{\circ }\left(e_{i},e_{j}\right)\right). \end{array}$$

Let K(π) denote the sectional curvature of a Riemannian manifold N of the plane section $\pi \subset T_{p}N$ at a point p∈N. Then,

(20) $$\begin{array}{c} \tau \left(p\right)=\sum_{1\leq i<j\leq n+1}K\left(e_{i}\wedge e_{j}\right), \end{array}$$

where τ is the scalar curvature. The normalized scalar curvature ρ is defined as

(21) $$\begin{array}{c} \rho =\frac{2\tau }{n(n+1)}. \end{array}$$

We also put

$$\zeta_{ij}^{\gamma }=g\left(\zeta \left(e_{i},e_{j}\right),e_{\gamma }\right),\;\zeta_{ij}^{*\gamma }=g\left(\zeta^{*}\left(e_{i},e_{j}\right),e_{\gamma }\right),$$

i,j∈1,2,…,n,n+1,γ∈{n+2,…,2m+1}. The squared norms of the second fundamental form ζ and $\zeta^{*}$ are denoted by C and $C^{*}$, respectively, and are given as

(22) $$\begin{array}{c} C=\frac{1}{n}\sum_{\gamma =n+2}^{2m+1}\sum_{i,j=1}^{n+1}{\left(\zeta_{ij}^{\gamma }\right)}^{2}\;\mathrm{and}\;C^{*}=\frac{1}{n}\sum_{\gamma =n+2}^{2m+1}\sum_{i,j=1}^{n+1}{\left(\zeta_{ij}^{*\gamma }\right)}^{2}, \end{array}$$

called Casorati curvatures of the submanifold [19,23].

Let $L_{r}$ be an *r*-dimensional subspace of TN, r≥2, and $\left\{e_{1},e_{2},\cdots ,e_{r}\right\}$ is an orthonormal basis of $L_{r}$. Then

$$\begin{array}{c} \tau \left(L_{r}\right)=\sum_{1\leq \gamma <\beta \leq r}K\left(e_{\gamma }\wedge e_{\beta }\right), \end{array}$$

is called the scalar curvature of the *r*-plane section. The Casorati curvatures C and $C^{*}$ of that *r*-plane section are [19,23]:

(23) $$\begin{array}{c} C\left(L_{r}\right)=\frac{1}{r}\sum_{\gamma =n+2}^{2m+1}\sum_{i,j=1}^{r}{\left(\zeta_{ij}^{\gamma }\right)}^{2}\;\mathrm{and}\;C^{*}\left(L_{r}\right)=\frac{1}{r}\sum_{\gamma =n+2}^{2m+1}\sum_{i,j=1}^{r}{\left(\zeta_{ij}^{*\gamma }\right)}^{2}. \end{array}$$

The normalized δ-Casorati curvatures $\delta_{c}\left(n\right)$ and ${\hat{\delta }}_{c}\left(n\right)$ are defined as [20,23]:

(24) $$\begin{array}{c} {\left[\delta_{c}\left(n\right)\right]}_{p}=\frac{1}{2}C_{p}+\frac{n+2}{2(n+1)}inf\left\{C\left(L_{n}\right)|L_{n}:a\;\mathrm{hyperplane}\;\mathrm{of}\;T_{p}N\right\} \end{array}$$

and

(25) $$\begin{array}{c} {\left[{\hat{\delta }}_{c}\left(n\right)\right]}_{p}=2C_{p}-\frac{2n+1}{2(n+1)}sup\left\{C\left(L_{n}\right)|L_{n}:a\;\mathrm{hyperplane}\;\mathrm{of}\;T_{p}N\right\}. \end{array}$$

Similarly, the dual normalized $\delta^{*}$-Casorati curvatures $\delta_{c}^{*}\left(n\right)$ and ${\hat{\delta }}_{c}^{*}\left(n\right)$ are defined as [14,19]:

(26) $$\begin{array}{c} {\left[\delta_{c}^{*}\left(n\right)\right]}_{p}=\frac{1}{2}C_{p}^{*}+\frac{n+2}{2(n+1)}inf\left\{C^{*}\left(L_{n}\right)|L_{n}:a\;\mathrm{hyperplane}\;\mathrm{of}\;T_{p}N\right\} \end{array}$$

and

(27) $$\begin{array}{c} {\left[{\hat{\delta }}_{c}^{*}\left(n\right)\right]}_{p}=2C_{p}^{*}-\frac{2n+1}{2(n+1)}sup\left\{C^{*}\left(L_{n}\right)|L_{n}:a\;\mathrm{hyperplane}\;\mathrm{of}\;T_{p}N\right\}. \end{array}$$

For a positive real number t≠n(n+1), put

(28) $$\begin{array}{c} b\left(t\right)=\frac{1}{(n+1)t}\left(n\right)\left(n+t+1\right)\left(n^{2}+n-t\right), \end{array}$$

then the generalized normalized δ-Casorati curvatures $\delta_{c}\left(t;n\right)$ and ${\hat{\delta }}_{c}\left(t;n\right)$ are given as [20,23]:

$$\begin{array}{c} {\left[\delta_{c}\left(t;n\right)\right]}_{p}=tC_{p}+b\left(t\right)inf\left\{C\left(L_{n}\right)|L_{n}:a\;\mathrm{hyperplane}\;\mathrm{of}\;T_{p}N\right\} \end{array}$$

if 0<t<n(n+1), and

$$\begin{array}{c} {\left[{\hat{\delta }}_{c}\left(t;n\right)\right]}_{p}=tC_{p}+b\left(t\right)sup\left\{C\left(L_{n}\right)|L_{n}:a\;\mathrm{hyperplane}\;\mathrm{of}\;T_{p}N\right\} \end{array}$$

if t>n(n+1).

Further, the dual generalized normalized $\delta^{*}$-Casorati curvatures $\delta_{c}^{*}\left(t;n\right)$ and ${\hat{\delta }}_{c}^{*}\left(t;n\right)$ are given as [14,19]:

$$\begin{array}{c} {\left[\delta_{c}^{*}\left(t;n\right)\right]}_{p}=tC_{p}^{*}+b\left(t\right)inf\left\{C^{*}\left(L_{n}\right)|L_{n}:a\;\mathrm{hyperplane}\;\mathrm{of}\;T_{p}N\right\} \end{array}$$

if 0<t<n(n+1), and

$$\begin{array}{c} {\left[{\hat{\delta }}_{c}^{*}\left(t;n\right)\right]}_{p}=tC_{p}^{*}+b\left(t\right)sup\left\{C^{*}\left(L_{n}\right)|L_{n}:a\;\mathrm{hyperplane}\;\mathrm{of}\;T_{p}N\right\} \end{array}$$

if t>n(n+1).

**Lemma** **1** (see Reference [**23**])**.** *Let*

$$F=\{\left(x_{1},\cdots ,x_{n+1}\right)\in R^{n+1}:x_{1}+\cdots +x_{n}+x_{n+1}=k\}$$

*be a hyperplane of $R^{n+1}$, and $f:R^{n+1}\to R$ a quadratic form given by*

$$\begin{array}{c} f\left(x_{1},\cdots ,x_{n+1}\right)=a\sum_{i=1}^{n}{\left(x_{i}\right)}^{2}+b{\left(x_{n+1}\right)}^{2}-2\sum_{1\leq i<j\leq n+1}x_{i}x_{j},\;a>0,b>0. \end{array}$$

*Then f has a global solution,*

(29) $$\begin{array}{c} \left\{\begin{array}{c} x_{1}=x_{2}=\cdots =x_{n}=\frac{1}{a+1}k,\\ x_{n+1}=\frac{1}{b+1}k, \end{array}\right. \end{array}$$

*provided*

$$b=\frac{n}{a-n+1}.$$

## 3. Normalized δ-Casorati Curvature

In this section, we mainly show that the normalized scalar curvature ρ is bounded above by the normalized δ-Casorati curvatures for statistical submanifolds of Sasaki-like statistical manifold with constant ϕ-sectional curvature.

**Theorem** **1.** Let N be a statistical submanifold in a Sasaki-like statistical manifold $\bar{N}\left(c\right)$ such that N is tangent to the structure vector field ξ of $\bar{N}\left(c\right)$. Then, the normalized δ-Casorati curvatures $\delta_{c}\left(n\right)$ and $\delta_{c}^{*}\left(n\right)$ satisfy

(30) $$\begin{array}{ccc} \rho & \leq & 2\delta_{c}^{\circ }\left(n\right)+\frac{c+3}{4}+\frac{c-1}{4n(n+1)}{\{\left(1-n\right)-\left(tr\phi \right)}^{2}+{2∥P∥}^{2}\}\\ & + & \frac{1}{n}C^{\circ }-\frac{n+1}{2n}{(∥H∥}^{2}+∥H^{*}{∥}^{2}), \end{array}$$

for real t, 0<t<n(n+1), where $2\delta_{c}^{\circ }\left(n\right)=\delta_{c}\left(n\right)+\delta_{c}^{*}\left(n\right)$, $2C^{\circ }=C+C^{*}$ and ${∥P∥}^{2}=g^{2}\left(\phi e_{j},e_{i}\right)$. The equality case holds in Equation (30) if and only if the component of ζ satisfies

(31) $$\left\{\begin{array}{l} \zeta_{11}^{\circ \gamma }=\zeta_{22}^{\circ \gamma }=\cdots =\zeta_{nn}^{\circ \gamma }=\frac{1}{2}\zeta_{n+1n+1}^{\circ \gamma },\;\alpha \in \left\{n+2,\cdots ,2m+1\right\},\\ \zeta_{ij}^{\circ \gamma }=0,\;i,j\in \left\{1,\cdots ,n+1\right\},\;\alpha \in \left\{n+2,\cdots ,2m+1\right\}. \end{array}\right.$$

**Proof.**  From Equations (7) and (12), we have

(32) $$\begin{array}{cc} g(R(X,Y)Z,W) & =\frac{c+3}{4}\{g\left(Y,Z\right)g\left(X,W\right)-g\left(X,Z\right)g\left(Y,W\right\}\\ & +\frac{c-1}{4}\{g\left(\phi Y,Z\right)g\left(\phi X,W\right)-g\left(\phi X,Z\right)g\left(\phi Y,W\right)\\ & -g(\phi X,Y)g(\phi Z,W)+g(X,\phi Y)g(\phi Z,W)\\ & -g(Y,\xi )g(Z,\xi )g(X,W)+g(X,\xi )g(Z,\xi )g(Y,W)\\ & +g(Y,\xi )g(Z,X)g(\xi ,W)-g(X,\xi )g(Z,Y)g(\xi ,W)\}\\ & -g\left(\zeta \left(X,Z\right),\zeta^{*}\left(Y,W\right)\right)+g\left(\zeta^{*}\left(X,W\right),\zeta \left(Y,Z\right)\right). \end{array}$$

Putting $Y=W=e_{i}$ and $X=Z=e_{j}$ in Equation (32), we get

(33) $$\begin{array}{cc} g(R\left(e_{i},e_{j}\right)e_{j},e_{i}) & =\frac{c+3}{4}\left\{g\left(e_{j},e_{j}\right)g\left(e_{i},e_{i}\right)-g\left(e_{i},e_{j}\right)g\left(e_{j},e_{i}\right)\right\}\\ & +\frac{c-1}{4}\{g\left(\phi e_{j},e_{j}\right)g\left(\phi e_{i},e_{i}\right)-g\left(\phi e_{i},e_{j}\right)g\left(\phi e_{j},e_{i}\right)\\ & -g\left(\phi e_{i},e_{j}\right)g\left(\phi e_{j},e_{i}\right)+g\left(e_{i},\phi e_{j}\right)g\left(\phi e_{j},e_{i}\right)\\ & -g\left(e_{j},\xi \right)g\left(e_{j},\xi \right)g\left(e_{i},e_{i}\right)+g\left(e_{i},\xi \right)g\left(e_{j},\xi \right)g\left(e_{j},e_{i}\right)\\ & +g\left(e_{j},\xi \right)g\left(e_{j},e_{i}\right)g\left(\xi ,e_{i}\right)-g\left(e_{i},\xi \right)g\left(e_{j},e_{j}\right)g\left(\xi ,e_{i}\right)\}\\ & -g\left(\zeta \left(e_{i},e_{j}\right),\zeta^{*}\left(e_{j},e_{i}\right)\right)+g\left(\zeta^{*}\left(e_{i},e_{i}\right),\zeta \left(e_{j},e_{j}\right)\right). \end{array}$$

Applying summation 1≤i,j≤n+1 and using Equations (14)–(19) in Equation (33), we obtain

(34) $$\begin{array}{cc} \sum_{1\leq i,j\leq n+1}g\left(R\left(e_{i},e_{j}\right)e_{j},e_{i}\right) & =\frac{c+3}{4}n\left(n+1\right)\\ & +\frac{c-1}{4}\left\{\left(1-n\right)-{\left(tr\phi \right)}^{2}+2g^{2}\left(\phi e_{j},e_{i}\right)\right\}\\ & +n^{2}g\left(H,H^{*}\right)-g\left(\zeta \left(e_{i},e_{j}\right),\zeta^{*}\left(e_{j},e_{i}\right)\right)\\ & =\frac{c+3}{4}n\left(n+1\right)\\ & +\frac{{\left(n+1\right)}^{2}}{2}\left\{g\left(H^{*}+H,H^{*}+H\right)-g\left(H,H\right)-g\left(H^{*},H^{*}\right)\right\}\\ & +\frac{c-1}{4}\left\{\left(1-n\right)-{\left(tr\phi \right)}^{2}+2g^{2}\left(\phi e_{j},e_{i}\right)\right\}\\ & -\frac{1}{2}\{g\left(\zeta \left(e_{i},e_{j}\right)+\zeta^{*}\left(e_{j},e_{i}\right),\zeta^{*}\left(e_{i},e_{j}\right)+\zeta \left(e_{j},e_{i}\right)\right)\\ & -g\left(\zeta \left(e_{i},e_{j}\right),\zeta \left(e_{i},e_{j}\right)\right)-g\left(\zeta^{*}\left(e_{j},e_{i}\right),\zeta^{*}\left(e_{j},e_{i}\right)\right)\}. \end{array}$$

Indeed, from Equation (2), $2H^{\circ }=H+H^{*}$. Then, from Equations (22) and (34) we see that

(35) $$\begin{array}{ccc} 2\tau & = & \frac{c+3}{4}n\left(n+1\right)+\frac{c-1}{4}\left\{\left(1-n\right)-{\left(tr\phi \right)}^{2}+2{∥P∥}^{2}\right\}\\ & + & 2{\left(n+1\right)}^{2}∥H^{\circ }{∥}^{2}-\frac{{\left(n+1\right)}^{2}}{2}{(∥H∥}^{2}+∥H^{*}{∥}^{2})\\ & - & 2\left(n+1\right)C^{\circ }+\frac{\left(n+1\right)}{2}\left(C+C^{*}\right). \end{array}$$

Now we write a quadratic polynomial Q as

(36) $$\begin{array}{cc} Q & =n\left(n+1\right)C^{\circ }+n\left(n+2\right)C^{\circ }\left(L_{n}\right)+\frac{c+3}{4}n\left(n+1\right)\\ & +\frac{c-1}{4}\left\{\left(1-n\right)-{\left(tr\phi \right)}^{2}+2{∥P∥}^{2}\right\}-2\tau +\frac{\left(n+1\right)}{2}\left(C+C^{*}\right)\\ & -\frac{{\left(n+1\right)}^{2}}{2}{(∥H∥}^{2}+∥H^{*}{∥}^{2}), \end{array}$$

where $L_{n}$ is the hyperplane of $T_{p}M$. Without loss of generality, let us assume that $L_{n}$ is spanned by $e_{1},\cdots ,e_{n}$, then Equation (36) yields

$$\begin{array}{ccc} Q & = & 2\left(n+1\right)\sum_{\gamma =n+2}^{2m+1}\sum_{i=1}^{n}{\left(\zeta_{ii}^{\circ \gamma }\right)}^{2}+4\left(n+2\right)\sum_{\gamma =n+2}^{2m+1}\sum_{i<j=1}^{n}{\left(\zeta_{ij}^{\circ \gamma }\right)}^{2}\\ & + & n\sum_{\gamma =n+2}^{2m+1}{\left(\zeta_{n+1n+1}^{\circ \gamma }\right)}^{2}+2\left(n+2\right)\sum_{\gamma =n+2}^{2m+1}\sum_{i=1}^{n}{\left(\zeta_{in}^{\circ \gamma }\right)}^{2}-4\sum_{\gamma =n+2}^{2m+1}\sum_{i<j=1}^{n+1}\zeta_{ii}^{\circ \gamma }\zeta_{jj}^{\circ \gamma }, \end{array}$$

which implies

(37) $$\begin{array}{ccc} \frac{1}{2}Q & = & \left(n+1\right)\sum_{\gamma =n+2}^{2m+1}\sum_{i=1}^{n}{\left(\zeta_{ii}^{\circ \gamma }\right)}^{2}+2\left(n+2\right)\sum_{\gamma =n+2}^{2m+1}\sum_{i<j=1}^{n}{\left(\zeta_{ij}^{\circ \gamma }\right)}^{2}\\ & + & \frac{n}{2}\sum_{\gamma =n+2}^{2m+1}{\left(\zeta_{n+1n+1}^{\circ \gamma }\right)}^{2}+\left(n+2\right)\sum_{\gamma =n+2}^{2m+1}\sum_{i=1}^{n}{\left(\zeta_{in}^{\circ \gamma }\right)}^{2}-2\sum_{\gamma =n+2}^{2m+1}\sum_{i<j=1}^{n+1}\zeta_{ii}^{\circ \gamma }\zeta_{jj}^{\circ \gamma }\\ & \geq & \sum_{\gamma =n+2}^{2m+1}\left[\left(n+1\right)\sum_{i=1}^{n}{\left(\zeta_{ii}^{\circ \gamma }\right)}^{2}+\frac{n}{2}{\left(\zeta_{n+1n+1}^{\circ \gamma }\right)}^{2}-2\sum_{i<j=1}^{n+1}\zeta_{ii}^{\circ \gamma }\zeta_{jj}^{\circ \gamma }\right]. \end{array}$$

Now, we consider the quadratic forms $f_{\gamma }:R^{n+1}\to R$ such that

(38) $$\begin{array}{ccc} f_{\gamma }(\zeta_{11}^{\circ \gamma },\zeta_{22}^{\circ \gamma }, & \cdots , & \zeta_{n+1n+1}^{\circ \gamma })\\ & = & \sum_{\gamma =n+2}^{2m+1}\left[\left(n+1\right)\sum_{i=1}^{n}{\left(\zeta_{ii}^{\circ \gamma }\right)}^{2}+\frac{n}{2}{\left(\zeta_{n+1n+1}^{\circ \gamma }\right)}^{2}-2\sum_{i<j=1}^{n+1}\zeta_{ii}^{\circ \gamma }\zeta_{jj}^{\circ \gamma }\right]. \end{array}$$

We start with the problem

$$\begin{array}{c} minf_{\gamma },\;\mathrm{subject}\;\mathrm{to}\;\Gamma :\zeta_{11}^{\circ \gamma }+\zeta_{22}^{\circ \gamma }+\cdots +\zeta_{n+1n+1}^{\circ \gamma }=k^{\gamma }, \end{array}$$

where $k^{\gamma }$ is a real constant. By comparing Equation (37) and Lemma 1, we get that

$$\begin{array}{c} a=n+1,\;b=\frac{n}{2}. \end{array}$$

Hence, a critical point of the problem has the following form:

(39) $$\left\{\begin{array}{c} \zeta_{11}^{\circ \gamma }=\zeta_{22}^{\circ \gamma }=\cdots =\zeta_{nn}^{\circ \gamma }=\frac{1}{n+2}k^{\gamma },\\ \zeta_{n+1n+1}^{\circ \gamma }=\frac{2}{n+2}k^{\gamma }. \end{array}\right.$$

Thus, we get

Q≥0,

which implies

(40) $$\begin{array}{ccc} 2\tau & \leq & n\left(n+1\right)C^{\circ }+n\left(n+2\right)C^{\circ }\left(L_{n}\right)+\frac{c+3}{4}n\left(n+1\right)\\ & + & \frac{c-1}{4}\left\{\left(1-n\right)-{\left(tr\phi \right)}^{2}+2{∥P∥}^{2}\right\}+\frac{\left(n+1\right)}{2}\left(C+C^{*}\right)\\ & - & \frac{{\left(n+1\right)}^{2}}{2}{(∥H∥}^{2}+∥H^{*}{∥}^{2}). \end{array}$$

From this, it follows that

$$\begin{array}{ccc} \rho & \leq & C^{\circ }+\frac{n+2}{n+1}C^{\circ }\left(L_{n}\right)+\frac{c+3}{4}\\ & + & \frac{c-1}{4n(n+1)}\left\{\left(1-n\right)-{\left(tr\phi \right)}^{2}+2{∥P∥}^{2}\right\}+\frac{1}{2n}\left(C+C^{*}\right)\\ & - & \frac{\left(n+1\right)}{2n}{(∥H∥}^{2}+∥H^{*}{∥}^{2})\\ & = & 2\delta_{c}^{\circ }\left(n\right)+\frac{c+3}{4}+\frac{c-1}{4n(n+1)}{\{\left(1-n\right)-\left(tr\phi \right)}^{2}+{2∥P∥}^{2}\}\\ & + & \frac{1}{2n}\left(C+C^{*}\right)-\frac{n+1}{2n}{(∥H∥}^{2}+∥H^{*}{∥}^{2}), \end{array}$$

which is the required inequality, and the equality in Equation (30) holds if and only if we have the equality in the all the previous inequalities. Thus, the equality holds in Equation (30) if and only if the relations in Equation (31) are true. ☐

**Remark** **2.** A similar result can also be obtained for normalized δ-Casorati curvatures ${\hat{\delta }}_{c}\left(n\right)$ and ${\hat{\delta }}_{c}^{*}\left(n\right)$.

## 4. Generalized Normalized δ-Casorati Curvature

In this section, we mainly show that the normalized scalar curvature is bounded above by the generalized normalized δ-Casorati curvatures for statistical submanifolds of Sasaki-like statistical manifold with constant ϕ-sectional curvature. We mainly prove the following result.

**Theorem** **2.** Let N be a statistical submanifold in a Sasaki-like statistical manifold $\bar{N}\left(c\right)$ such that N is tangent to the structure vector field ξ of $\bar{N}\left(c\right)$. Then, the generalized normalized δ-Casorati curvatures $\delta_{c}\left(t;n\right)$ and $\delta_{c}^{*}\left(t;n\right)$ satisfy

(41) $$\begin{array}{ccc} \rho & \leq & \frac{2}{n(n+1)}\delta_{c}^{\circ }\left(t;n\right)+\frac{c+3}{4}+\frac{c-1}{4n(n+1)}{\{\left(1-n\right)-\left(tr\phi \right)}^{2}+{2∥P∥}^{2}\}\\ & + & \frac{1}{n}C^{\circ }-\frac{n+1}{2n}{(∥H∥}^{2}+∥H^{*}{∥}^{2}), \end{array}$$

for real t, 0<t<n(n+1), where $2\delta_{c}^{\circ }\left(t;n\right)=\delta_{c}\left(t;n\right)+\delta_{c}^{*}\left(t;n\right)$, $2C^{\circ }=C+C^{*}$ and ${∥P∥}^{2}=g^{2}\left(\phi e_{j},e_{i}\right)$. The equality case holds in Equation (41) if and only if the component of ζ satisfies

(42) $$\left\{\begin{array}{c} \zeta_{ij}^{\circ \gamma }=0,\;i,j\in \left\{1,\cdots ,n+1\right\},\;\alpha \in \left\{n+2,\cdots ,2m+1\right\},\\ \zeta_{11}^{\circ \gamma }=\zeta_{22}^{\circ \gamma }=\cdots =\zeta_{nn}^{\circ \gamma }=\frac{t}{n(n+1)}\zeta_{n+1n+1}^{\circ \gamma },\;\alpha \in \left\{n+2,\cdots ,2m+1\right\}. \end{array}\right.$$

**Proof.**  Keeping in mind the scalar curvature in Equation (35), we may assume a quadratic polynomial T as

(43) $$\begin{array}{cc} T & =2tC^{\circ }+2b\left(t\right)C^{\circ }\left(L_{n}\right)+\frac{c+3}{2}n\left(n+1\right)+\frac{c-1}{4}\left\{\left(1-n\right)-{\left(tr\phi \right)}^{2}+2{∥P∥}^{2}\right\}\\ & -2\tau +\frac{\left(n+1\right)}{2}\left(C+C^{*}\right)-\frac{{\left(n+1\right)}^{2}}{2}{(∥H∥}^{2}+∥H^{*}{∥}^{2}), \end{array}$$

where $L_{n}$ is the hyperplane of $T_{p}M$. Without loss of generality, let us assume that $L_{n}$ is spanned by $e_{1},\cdots ,e_{n}$, then from Equation (43) it follows that

$$\begin{array}{ccc} T & = & 2\left(\frac{t}{n+1}+\frac{b(t)}{n}\right)\sum_{\gamma =n+2}^{2m+1}\sum_{i=1}^{n}{\left(\zeta_{ii}^{\circ \gamma }\right)}^{2}\\ & + & 4\left(\frac{t}{n+1}+\frac{b(t)}{n}+1\right)\sum_{\gamma =n+2}^{2m+1}\sum_{i<j=1}^{n}{\left(\zeta_{ij}^{\circ \gamma }\right)}^{2}+\left(\frac{2t}{n+1}\right)\sum_{\gamma =n+2}^{2m+1}{\left(\zeta_{n+1n+1}^{\circ \gamma }\right)}^{2}\\ & + & 4\left(\frac{t}{n+1}+1\right)\sum_{\gamma =n+2}^{2m+1}\sum_{i=1}^{n}{\left(\zeta_{in}^{\circ \gamma }\right)}^{2}-4\sum_{\gamma =n+2}^{2m+1}\sum_{i<j=1}^{n+1}\zeta_{ii}^{\circ \gamma }\zeta_{jj}^{\circ \gamma }, \end{array}$$

which implies

(44) $$\begin{array}{ccc} \frac{1}{2}T & = & \left(\frac{t}{n+1}+\frac{b(t)}{n}\right)\sum_{\gamma =n+2}^{2m+1}\sum_{i=1}^{n}{\left(\zeta_{ii}^{\circ \gamma }\right)}^{2}\\ & + & 2\left(\frac{t}{n+1}+\frac{b(t)}{n}+1\right)\sum_{\gamma =n+2}^{2m+1}\sum_{i<j=1}^{n}{\left(\zeta_{ij}^{\circ \gamma }\right)}^{2}+\left(\frac{t}{n+1}\right)\sum_{\gamma =n+2}^{2m+1}{\left(\zeta_{n+1n+1}^{\circ \gamma }\right)}^{2}\\ & + & 2\left(\frac{t}{n+1}+1\right)\sum_{\gamma =n+2}^{2m+1}\sum_{i=1}^{n}{\left(\zeta_{in}^{\circ \gamma }\right)}^{2}-2\sum_{\gamma =n+2}^{2m+1}\sum_{i<j=1}^{n+1}\zeta_{ii}^{\circ \gamma }\zeta_{jj}^{\circ \gamma }\\ & \geq & \sum_{\gamma =n+2}^{2m+1}[\left(\frac{t}{n+1}+\frac{b(t)}{n}\right)\sum_{i=1}^{n}{\left(\zeta_{ii}^{\circ \gamma }\right)}^{2}\\ & + & \left(\frac{t}{n+1}\right){\left(\zeta_{n+1n+1}^{\circ \gamma }\right)}^{2}-2\sum_{i<j=1}^{n+1}\zeta_{ii}^{\circ \gamma }\zeta_{jj}^{\circ \gamma }]. \end{array}$$

Now, we consider the quadratic forms $f_{\gamma }:R^{n+1}\to R$ such that

(45) $$\begin{array}{ccc} f_{\gamma }\left(\zeta_{11}^{\circ \gamma },\zeta_{22}^{\circ \gamma },\cdots ,\zeta_{n+1n+1}^{\circ \gamma }\right) & = & \sum_{\gamma =n+2}^{2m+1}[\left(\frac{t}{n+1}+\frac{b(t)}{n}\right)\sum_{i=1}^{n}{\left(\zeta_{ii}^{\circ \gamma }\right)}^{2}\\ & + & \left(\frac{t}{n+1}\right){\left(\zeta_{n+1n+1}^{\circ \gamma }\right)}^{2}-2\sum_{i<j=1}^{n+1}\zeta_{ii}^{\circ \gamma }\zeta_{jj}^{\circ \gamma }]. \end{array}$$

We start with the problem

$$\begin{array}{c} minf_{\gamma },\;\mathrm{subject}\;\mathrm{to}\;\Gamma :\zeta_{11}^{\circ \gamma }+\zeta_{22}^{\circ \gamma }+\cdots +\zeta_{n+1n+1}^{\circ \gamma }=k^{\gamma }, \end{array}$$

where $k^{\gamma }$ is a real constant.

By comparing Equation (44) and Lemma 1, it is easy to see that

$$\begin{array}{c} a=\frac{t}{n+1}+\frac{b(t)}{n},\;b=\frac{t}{n+1}. \end{array}$$

Hence, a critical point of the problem has the following form:

(46) $$\left\{\begin{array}{c} \zeta_{11}^{\circ \gamma }=\zeta_{22}^{\circ \gamma }=\cdots =\zeta_{nn}^{\circ \gamma }=\frac{1}{\frac{t}{n+1}+\frac{b(t)}{n}+1}k^{\gamma }\\ \zeta_{n+1n+1}^{\circ \gamma }=\frac{1}{\frac{t}{n+1}+1}k^{\gamma }. \end{array}\right.$$

Thus, we get

T≥0,

which implies

(47) $$\begin{array}{ccc} 2\tau & \leq & 2tC^{\circ }+2b\left(t\right)C^{\circ }\left(L_{n}\right)+\frac{c+3}{4}n\left(n+1\right)\\ & + & \frac{c-1}{4}\left\{\left(1-n\right)-{\left(tr\phi \right)}^{2}+2{∥P∥}^{2}\right\}+\frac{\left(n+1\right)}{2}\left(C+C^{*}\right)\\ & - & \frac{{\left(n+1\right)}^{2}}{2}{(∥H∥}^{2}+∥H^{*}{∥}^{2}). \end{array}$$

From this it follows that

$$\begin{array}{ccc} \rho & \leq & \frac{2t}{n(n+1)}C^{\circ }+\frac{2b(t)}{n(n+1)}C^{\circ }\left(L_{n}\right)+\frac{c+3}{4}\\ & + & \frac{c-1}{4n(n+1)}\left\{\left(1-n\right)-{\left(tr\phi \right)}^{2}+2{∥P∥}^{2}\right\}+\frac{1}{2n}\left(C+C^{*}\right)\\ & - & \frac{\left(n+1\right)}{2n}{(∥H∥}^{2}+∥H^{*}{∥}^{2})\\ & = & \frac{2}{n(n+1)}\delta_{c}^{\circ }\left(t;n\right)+\frac{c+3}{4}+\frac{c-1}{4n(n+1)}{\{\left(1-n\right)-\left(tr\phi \right)}^{2}+{2∥P∥}^{2}\}\\ & + & \frac{1}{2n}\left(C+C^{*}\right)-\frac{n+1}{2n}{(∥H∥}^{2}+∥H^{*}{∥}^{2}), \end{array}$$

which is the required inequality. The equality in Equation (41) holds if and only if we have the equality in the all the previous inequalities. Thus, the equality holds in Equation (41) if and only if the relations in Equation (42) are true. ☐

**Remark** **3.** A similar result can also be obtained for generalized normalized δ-Casorati curvatures ${\hat{\delta }}_{c}\left(t;n\right)$ and ${\hat{\delta }}_{c}^{*}\left(t;n\right)$.

## 5. η-Einstein Sasaki-Like Statistical Manifolds

In 1962, Okumura [22] introduced and studied the η-Einstein manifold. In 1965, Sasaki [24] named it η-Einstein. Since then a number of papers have been published on this topic due to its application to the physics or in particular to the theory of relativity.

In this section, we obtain the following results.

**Theorem** **3.** *A Sasaki-like statistical manifold $\bar{N}\left(c\right)$ is η-Einstein if and only if trϕ=0. Moreover, μ and ν are constants and are equal to*

$$\begin{array}{c} \mu =\frac{1}{4}\left(2nc+6n+3c-3\right),\\ \nu =\frac{1-c}{4}\left(2n+1\right). \end{array}$$

**Proof.**  Taking the inner product of Equation (12) with W, we find

(48) $$\begin{array}{cc} g(\bar{R}\left(X,Y\right)Z,W) & =\frac{c+3}{4}\left\{g\left(Y,Z\right)g\left(X,W\right)-g\left(X,Z\right)g\left(Y,W\right)\right\}\\ & +\frac{c-1}{4}\{g\left(\phi Y,Z\right)g\left(\phi X,W\right)-g\left(\phi X,Z\right)g\left(\phi Y,W\right)\\ & -2g(\phi X,Y)g(\phi Z,W)-g(Y,\xi )g(Z,\xi )g(X,W)\\ & +g(X,\xi )g(Z,\xi )g(Y,W)+g(Y,\xi )g(Z,X)g(\xi ,W)\\ & -g(X,\xi )g(Z,Y)g(\xi ,W)\}. \end{array}$$

Substituting $X=W=e_{i}$ in Equation (48) and taking summation 1≤i≤2n+1, we get

(49) $$\begin{array}{cc} \sum_{i=1}^{2n+1}g\left(\bar{R}\left(e_{i},Y\right)Z,e_{i}\right) & =\frac{c+3}{4}\sum_{i=1}^{2n+1}\left\{g\left(Y,Z\right)g\left(e_{i},e_{i}\right)-g\left(e_{i},Z\right)g\left(Y,e_{i}\right)\right\}\\ & +\frac{c-1}{4}\sum_{i=1}^{2n+1}\{g\left(\phi Y,Z\right)g\left(\phi e_{i},e_{i}\right)-g\left(\phi e_{i},Z\right)g\left(\phi Y,e_{i}\right)\\ & -2g\left(\phi e_{i},Y\right)g\left(\phi Z,e_{i}\right)-g\left(Y,\xi \right)g\left(Z,\xi \right)g\left(e_{i},e_{i}\right)\}, \end{array}$$

which implies

(50) $$\begin{array}{cc} S(Y,Z) & =\frac{1}{4}\left(2nc+6n+3c-3\right)g\left(Y,Z\right)+\frac{c-1}{4}g\left(\phi Y,Z\right)\left(tr\phi \right)\\ & -\frac{c-1}{4}\left(2n+1\right)\eta \left(Y\right)\eta \left(Z\right). \end{array}$$

With trϕ=0, the above equation takes the following form:

(51) $$\begin{array}{cc} S(Y,Z) & =\frac{1}{4}\left(2nc+6n+3c-3\right)g\left(Y,Z\right)-\frac{c-1}{4}\left(2n+1\right)\eta \left(Y\right)\eta \left(Z\right). \end{array}$$

Hence, $\bar{N}\left(c\right)$ is η-Einstein with $\mu =\frac{1}{4}\left(2nc+6n+3c-3\right)$ and $\nu =\frac{1-c}{4}\left(2n+1\right)$. A straight-forward computation proves the converse part. ☐

**Theorem** **4.** *The Ricci curvature tensor of η-Einstein Sasaki-like statistical manifold $\bar{N}\left(c\right)$ in the direction of ξ is*

(52) $$\begin{array}{c} \frac{1}{4}\left(5c+8n+1\right). \end{array}$$

**Proof.**  Setting Y=Z=ξ in Equation (51), we obtain the required result. ☐

**Theorem** **5.** *If the Ricci curvature tensor of an η-Einstein Sasaki-like statistical manifold $\bar{N}\left(c\right)$ in the direction of ξ, then*

(53) $$\begin{array}{c} \mu =\frac{r}{2n}-\frac{5c+8n+1}{8n}, \end{array}$$

(54) $$\begin{array}{c} \nu =r-\left(2n-1\right)\left[\frac{r}{2n}-\frac{5c+8n+1}{8n}\right]. \end{array}$$

**Proof.**  From Equation (13) we have

(55) $$\begin{array}{c} S(\xi ,\xi )=\mu +\nu . \end{array}$$

Also, from Equation (51), we get

(56) $$\begin{array}{c} S\left(\xi ,\xi \right)=\frac{1}{4}\left(5c+8n+1\right). \end{array}$$

Combining Equations (55) and (56), we find

(57) $$\begin{array}{c} \mu +\nu =\frac{1}{4}\left(5c+8n+1\right). \end{array}$$

Further, from Equation (13), we obtain

(58) $$\begin{array}{c} r=(2n+1)\mu +\nu . \end{array}$$

Solving Equations (57) and (58) for μ and ν, we have the required result. ☐

**Theorem** **6.** *The scalar curvature of an η-Einstein Sasaki-like statistical manifold $\bar{N}\left(c\right)$ is constant and equal to*

(59) $$\begin{array}{c} \frac{1}{4}\left(2n+1\right)\left(2nc+5c+6n+1\right). \end{array}$$

**Proof.**  Putting $Y=Z=e_{i}$ in Equation (51) and taking summation 1≤i≤2n+1, we have our assertion. ☐

We give an example of Theorem 3, which is the following:

**Example** **5.** *We recall from Example 2.2 of Reference [4] that $\left(\bar{N}=\left\{\left(x^{1},\cdots ,x^{m},y^{1},\cdots ,y^{m},z\right)\in R^{2m+1}\right\},\bar{\nabla },g,\phi ,\xi ,\eta \right)$ is a Sasaki-like statistical manifold with c=−3 and the structure tensors (g,ϕ,ξ,η) are defined by*

$$\begin{array}{c} g=\left(\begin{array}{ccc} 2\delta_{ij}+y^{i}y^{j} & 0 & -y^{i}\\ 0 & -\delta_{ij} & 0\\ -y^{i} & 0 & 1 \end{array}\right),\;\phi =\left(\begin{array}{ccc} 0 & \delta_{ij} & 0\\ -\delta_{ij} & 0 & 0\\ 0 & y^{j} & 0 \end{array}\right),\\ \xi =\left(\begin{array}{c} 0\\ \vdots \\ 0\\ 1 \end{array}\right),\;\eta =\left(-y^{1},0,-y^{2},0,\cdots ,-y^{m},0,1\right). \end{array}$$

*We see that trace(ϕ)=0. Thus, by Theorem 3, we conclude that ${\bar{N}}^{2m+1}\left(-3\right)$ is an η-Einstein manifold.*

Theorem 3 yields the following corollary.

**Corollary** **1.** *The η-Einstein Sasaki-like statistical manifold $\bar{N}\left(c\right)$ becomes Einstein if c=1. Moreover, in that case μ=2n.*

**Proof.**  One can easily obtain the result by just substituting c=1 in Equation (51). ☐

## 6. Conclusions

We have the following conclusions from this work:

1. By using a different approach, we obtained a relationship between a new extrinsic invariant called the Casorati curvature and an intrinsic invariant called the normalized scalar curvature of statistical manifolds with any co-dimension of Sasaki-like statistical space forms. The derived relations can motivate other researchers to obtain similar relationships for many kinds of invariants of similar nature, for statistical submanifolds in different ambient spaces, such as Kaehler-like statistical manifolds, Kenmotsu-like statistical manifolds, cosymplectic-like statistical manifolds, and statistical warped product manifolds.
2. An Einstein Sasaki-like statistical manifold can not be Ricci-flat.
3. The metric of the Sasaki-like statistical manifolds with constant curvature is a solution of the vacuum Einstein field equation if the manifold is η-Einstein with constant curvature c=1. In fact, the Einstein field equations consist of 10 equations in Einstein’s general theory of relativity. This theory tells us the fundamental interaction of gravitation. Actually, the Einstein field equations are used to obtain the spacetime geometry which are the the outcome of the presence of linear momentum and mass-energy. Therefore, it is of great interest to see what type of solution we can obtain for Einstein field equations in the case of the metric of Sasaki-like statistical manifold.
